# Pro-Inflammatory Nutrient: Focus on Gliadin and Celiac Disease

**DOI:** 10.3390/ijms23105577

**Published:** 2022-05-17

**Authors:** Maria Vittoria Barone, Auricchio Salvatore

**Affiliations:** ELFID (European Laboratory for the Investigation of Food Induced Diseases), Department of Translational Medical Science, University of Naples, Federico II, 80131 Naples, Italy

Ingested food can cause tissue inflammation through different mechanisms. Nutrient surplus, for example, can trigger intracellular stress signals that potentiate pro-inflammatory signaling. In general, a western diet and lifestyle have been long linked to low grade inflammation that represent the “common background” of several different diseases. Interestingly, the intestine appears to be a crossroad for the control of the inflammation, both locally and at a distance. An example of an intestinal inflammatory and remodeling response of the intestine to food is the small intestinal lesion in celiac disease (CD), induced by gluten—an alimentary protein present in wheat and other cereals.

A central role in the pathogenesis of CD is played by the HLA-restricted gliadin-specific intestinal T cell response. Although the activation of the T cells has been studied in depth, the central question remains still unanswered, namely, why a pro-inflammatory T cell response is generated towards gliadin instead of a regulatory response, which normally promotes oral tolerance to dietary protein antigens. At the same time, when, how, and where this inflammation is generated in CD is still not clear.

The recent literature describes in CD a meeting of several different factors, such as cellular vulnerability, pro-inflammatory effects of gluten and other wheat proteins, and a western diet and other environmental triggers, such as viruses, that prepare, and/or amplify, the T cell-mediated response to gluten. On one side, there is the pro-inflammatory environment (exogenous stimuli), such as diet, viruses, and other pro-inflammatory factors, and on the other side, there are the constitutive cellular alterations (endogenous predisposition) that by themselves induce and/or render the cells more sensitive to pro-inflammatory stimuli. All these factors, both exogenous and endogenous, can contribute to the generation of a “sterile” inflammation in CD.

In this Special Issue, we have collected some papers focusing on the peculiar organoleptic characteristic of gliadin [1], and wheat proteins in general [2,3], on the attempts to prevent the meeting of the gliadin and the intestinal epithelium as a meaning to prevent the diseases [4], and on the constitutive alterations [5] distinguishing the CD cells that could render them more sensitive to inflammatory agents including viruses [6], gliadin itself, and more generally, the western diet and lifestyle.

Gliadin, the main protein contained in wheat and other cereals, is a peculiar protein that contains long stretches of glutamine and proline. Due to its amino acids sequence and structural characteristics, it is difficult to digest by the human intestinal endopeptidases. The peptides that remain undigested can have different biological activities. Some of these peptides are known to activate the T cell response, whereas some others, such the peptide P31–43, does not activate the T cell response but has pleiotropic biological activity. Falcigno et al. [1], in this Special Issue, have studied the structural reasons why some gluten peptides prompt the adaptive immune systems while others do not, by apparently involving just the innate immune routes. Using several different approaches, they conclude that P31–43 is a non-adaptive prompter because it is not a good ligand for HLA-DQ. Even sharing a similar ability to adopt the polyproline II structure with the adaptive ones, the way in which the proline residues are located along the sequence disfavors a productive P31–43-HLA-DQ binding.

The special features of the wheat gluten prompted the research of wheat cultivars that could contain less toxic peptides. Among this field of research, the paper by Truzzi et al. [2] published in this Special Issue offers a good example. In fact, in this manuscript, the authors have made two main points. One is about methods to study the wheat toxicity in pre-clinical studies. The absence of an animal model in CD forced the researchers in this field to find alternative ways to study the disease. Cellular models, such as secondary or primary intestinal cells, or intestinal biopsies cultures, are, at this point, the best substitute of the animal models for this disease. Caco-2 cells, an intestinal secondary cell line derived from colon carcinoma, is responsive to gliadin and gliadin peptides. Truzzi et al. [2] used Caco-2 cells in 3D cultures, together with an extra-cellular matrix containing U937 monocytes and L929 fibroblasts, resembling all the main cellular structures present in the intestine. By the use of this 3D model, they concluded that the modern wheat proteins, due to gluten polymorphisms and increased gluten strength, are more cytotoxic and immunogenic than the landrace wheat proteins.

As a way to reduce wheat toxicity, nutraceuticals or probiotics have been extensively used in CD. Several clinical trials, mostly ongoing, have focused on targeting the immune response or gluten proteins by methods to induce immunosuppression and enhanced protein degradation by bacteria and protein sequestration. In this Special Issue, the paper by Van Buiten C and Elias R [4] extensively reviews one of the latest methods to reduce wheat toxicity: the polyphenols. Recent studies suggest that polyphenols may elicit several protective effects in celiac disease by reducing the enzymatic hydrolysis of gluten proteins, sequestering gluten proteins and exerting a general anti-inflammatory effect. This review highlights mechanisms by which polyphenols can protect against celiac disease, takes a critical look at recent literature, and outlines future applications for this potential treatment method.

Different stimuli may contribute to the intestinal damage in CD. Some of them come from the environment and include different pro-inflammatory agents that can cooperate or potentiate the gliadin effects on cells, including viruses, diet and other environmental factors. Gliadin itself can induce in intestinal cells different biological effects including proliferation, activation of the innate immunity, and several different forms of cell death. The review by Perez F et al. [3] published in this Special Issue summarizes the recent literature addressing the role of programmed cell death pathways in the small intestine, describing how these mechanisms may contribute to CD. Interestingly, programmed cell dead seem to play a key role in inducing inflammation.

The viral infections are among the environmental factors that can contribute to or potentiate the gliadin effects on the intestine. In this Special Issue, a short commentary is present on this topic by Barone MV and Auricchio S [6] that summarizes the recent literature on the possible combined role of viruses and food as triggers for CD. Various experimental and clinical observations suggest that multiple agents, such as viruses and bacteria, have some common inflammatory pathways predisposing individuals to chronic inflammatory diseases, including celiac disease (CD). A cooperation between viruses and gliadin is present in vitro and in vivo with common mechanisms to induce inflammation. These clinical and preclinical observations may have an impact on clinical practice in the management of patients at risk of CD.

Although some information is now available on the role of pro-inflammatory agents on CD intestine, it is still not clear why the CD intestine and cells are more sensitive to their effects. Some hypotheses have been put forward to try to respond to this question, but very likely the genetic/epigenetic background of CD cells has a key role. A manuscript by Discepolo et al. [5] presented in this Special Issue described structural differences between dendritic cells (DC) from CD with respect to controls. Moreover, this is one of the few reports that tries to link genetics and cellular phenotype in CD. In fact, the authors correlate recent genetic and expression studies to the DC shape. A role for genes involved in cell shape, adhesion, and actin rearrangements, including a Rho family regulator, Rho GTPase-activating protein 31 (ARHGAP31), has been previously demonstrated in CD. The authors found that ARHGAP31 is reduced in CD DCs, and that the Rho activity is reduced constitutively. Under these conditions, adhesion on fibronectin was able to discriminate the shape of CD patients from the control DCs, revealing a gluten-independent CD-specific cellular phenotype related to DC shape and regulated by RhoA activity.

We hope that the combination of review and original articles selected for this Special Issue could help to deepen the understanding of CD pathogenesis.
